# Cost-effectiveness of artificial intelligence interventions for musculoskeletal disorders of the spine: a systematic review

**DOI:** 10.1007/s00296-026-06122-3

**Published:** 2026-05-23

**Authors:** Tadesse Gebrye, Chidozie Mbada, Faatihah Niyi-Odumosu, Clara Fatoye, Marufat Odetunde, Zalmai Hakimi, Ushotanefe Useh, Francis Fatoye

**Affiliations:** 1 Department of Health Professions, Manchester Metropolitan University, Manchester, UK; 2 Lifestyle Diseases, Faculty of Health Sciences, North‒West University, Potchefstroom, South Africa; 3 School of Applied Sciences, University of the West of England, Bristol, UK; 4 Sobi AB, Stockholm, Sweden; 5 Department of Medical Rehabilitation, College of Health Sciences, Obafemi Awolowo University, Ile-Ife, Nigeria; 6 Department of Health Professions, Faculty of Health and Education, Manchester Metropolitan University, Brooks Building, 53 Bonsall Street, Manchester, M15 6GX UK

**Keywords:** Musculoskeletal diseases, Low back pain, Spine, Artificial intelligence, Machine learning, Cost-benefit analysis, Cost-effectiveness analysis

## Abstract

Musculoskeletal disorders (MSDs) represent a substantial global health burden, and artificial intelligence (AI)-based technologies are emerging as potential approaches for their management. However, evidence regarding the cost-effectiveness of these interventions remains limited. This systematic review synthesised the available evidence on the cost-effectiveness of AI-based technologies for managing MSDs of the spine. This systematic review was conducted and reported following the Preferred Reporting Items for Systematic Reviews and Meta-Analyses guideline. PubMed, Medline, CINAHL, Cochrane Central Register of Controlled Trials, Scopus, Web of Science, and Directory of Open Access Journals (DOAJ) databases were searched. After title and abstract screening, the remaining articles underwent full-text review by two independent reviewers, who extracted economic outcome data comparing AI-based interventions versus control. A total of 347 studies were identified, with 59 duplicates removed and 251 excluded based on title and abstract. After full-text review, seven eligible studies were included in the review. These studies represented five countries (United States (*n* = 2), Germany (*n* = 2), Denmark (*n* = 1), Australia (*n* = 1) and South Korea (*n* = 1)). The studies adopted a healthcare perspective (*n* = 4), a societal perspective (*n* = 2) and a healthcare and societal perspective (*n* = 1). Time durations varied widely, from four weeks to twelve months in trials to three years or lifetime in models, with one study using a 20-month post-implementation period. The AI components of the interventions encompassed a range of technologies, including a deep learning–based diagnostic and exercise prescription platform, AI-driven chest radiograph screening, the self-BACK application, standardised Magnetic Resonance Imaging (MRI) protocols, the Back Pain Choices online decision support tool, and the Kaia back pain application. In three studies, AI-based interventions for MSDs of the spine showed consistent positive QALY gains. Across the seven studies, five interventions (71%) were found to be cost-effective or cost-saving, four (57%) were dominant, while two (29%) were not cost-effective, indicating that most AI-based and digital interventions for MSDs of the spine provide favourable economic value compared with standard care. This is the first systematic review to synthesise evidence on the cost-effectiveness of AI-based technologies for the management of MSDs of the spine. The results suggest that AI-based interventions have the potential to contribute to more efficient healthcare resource use and may support policy and decision-making. However, their impact on reducing the economic burden of MSDs of the spine and improving health outcomes is not yet conclusive, and further standardised economic evaluations are needed to strengthen the evidence base.* PROSPERO registration number*: https://www.crd.york.ac.uk/PROSPERO/view/CRD42025642775.

**Supplementary Information:**

The online version contains supplementary material available at 10.1007/s00296-026-06122-3.

## Introduction

Musculoskeletal disorders (MSDs), particularly low back pain (LBP), remain among the most common causes of disability worldwide and represent a major public health and economic challenge [1]. MSDs are associated with reduced quality of life (QoL), impaired function, and contribute to significant productivity losses. The global one-year prevalence of LBP is estimated at 38%, with substantially higher rates reported in some high-income countries [2]. The economic burden associated with MSDs driven by healthcare costs, lost productivity, and long-term work disability is substantial, with LBP alone accounting for billions in direct and indirect costs each year across both high- and low-income settings [2].

Management of MSDs involves a mix of preventive, conservative, and rehabilitative strategies. While prevention includes ergonomic and educational interventions [3], conservative care is centred on pain relief through pharmacological management and physiotherapy approaches, such as exercise and manual therapy [4]. Rehabilitative strategies focus on restoring function through strength/endurance training and activity pacing [5]. Complementary methods, such as cognitive-behavioural therapy, address psychosocial factors [6], while assistive devices and workplace adaptations enhance long-term function. In severe cases, surgical options may be explored, followed by rehabilitation to improve outcomes [7].

Despite the availability of these interventions, care pathways for chronic LBP are often fragmented, involving repeated specialist consultations, delayed diagnoses, and inefficient use of healthcare resources. Recent socio-economic and clinical work has highlighted the potential of value-based, multidisciplinary, and lean healthcare models to address these challenges. In a university hospital setting, a lean, value-based multidisciplinary pathway integrating neurosurgical and pain management expertise within a single second-level consultation reduced unnecessary service use, shortened time to diagnosis, and improved patient-reported outcomes, with more than half of patients reporting substantial pain improvement. Importantly, this approach also demonstrated reductions in healthcare costs and productivity losses by avoiding redundant visits and delays in care, underscoring the importance of system-level innovation in chronic LBP management [8].

Furthermore, limited access to many interventions due to resource constraints and the high cost of alternative treatments has driven the emergence of AI-based approaches, including machine learning algorithms, computer vision, and wearable sensors, as innovative tools to support the management of MSDs [9]. AI-enabled approaches have attracted growing attention because of their scalability, personalisation, and real-time monitoring capabilities [10]. Machine learning models, such as convolutional neural networks and recurrent neural networks, can detect movement abnormalities, analyse kinematic patterns, and facilitate remote monitoring [11]. AI-enabled applications, including selfBACK [12] and the Kaia back pain application [13], have demonstrated potential benefits in improving pain, function, and self-management while supporting delivery of guideline-based care [14].

The rapid expansion of digital care models further highlighted the value of AI-enabled interventions in maintaining access to musculoskeletal care, particularly during periods when in-person services were constrained [15, 16]. Early randomised controlled trials of AI-assisted applications have demonstrated modest but statistically significant improvements in pain-related disability, with potential downstream implications for cost savings [17, 18].

Nevertheless, the cost-effectiveness of AI-based interventions for MSDs of the spine remains poorly understood. Existing economic evaluations suggest potential reductions in disability and healthcare utilisation; however, evidence remains fragmented and varies by analytic perspective, follow-up duration, and healthcare setting [13, 16, 18]. Many studies rely on short-term horizons, limiting insights into long-term sustainability and scalability. In addition, real-world implementation evidence remains scarce, making it challenging to determine system-level impacts, equity implications, and alignment with value-based healthcare principles demonstrated in multidisciplinary care models.

Given the substantial global clinical and economic burden of MSDs, this systematic review is the first to comprehensively synthesise evidence on the cost-effectiveness of AI-based interventions in rheumatology for MSDs of the spine, addressing a key gap in previous publications that have focused primarily on clinical effectiveness rather than economic value.

## Methods

### Protocol and reporting

This systematic review was conducted in accordance with the PRISMA 2020 statement. A protocol for this systematic review was prospectively registered on PROSPERO and can be found at https://www.crd.york.ac.uk/PROSPERO/view/CRD42025642775.

### Search strategy

We systematically searched PubMed, MEDLINE, CINAHL, Web of Science, and the Cochrane Central Register of Controlled Trials from database inception to 18th of November 2025. We additionally included the Scopus and Directory of Open Access Journals databases in our search strategy to ensure comprehensive coverage of the literature from inception through 20 April 2026. Search terms combined Medical Subject Headings (MeSH) and free-text words relating MSDs and artificial intelligence, including: machine learning, deep learning, clinical decision support system, natural language processing, computer-aided diagnosis, predictive analytics, robotic process automation, digital therapeutics, case-based reasoning, expert system, algorithmic diagnosis, virtual health assistant, chatbot, artificial intelligence, AI, cost-utility, cost-benefit, cost-effectiveness, cost-consequence, economic evaluation, economic modelling, economic assessment, back pain, neck pain, spinal pain, thoracic pain, cervical pain, lumbar pain, knee pain, shoulder pain, elbow pain, hand pain, wrist pain, ankle pain, foot pain, hip pain, and musculoskeletal pain. Search strategies were adapted for each database, and all terms were cross-checked against MeSH (Appendix 1). We also reviewed reference lists of eligible articles and relevant reviews. Abstracts from international conferences were screened but excluded if methodological detail was insufficient.

All records were imported into EndNote (Clarivate Analytics), and duplicates were removed. Two reviewers (TG and CM) independently screened titles and abstracts. Full-text articles were retrieved for potentially relevant studies. Discrepancies were resolved by discussion with a third reviewer (FNO).

### Eligibility criteria

The population of interest comprises adults (≥ 18 years) with MSDs, including non-specific or chronic low back pain, inflammatory back pain, degenerative spine disorders, and related musculoskeletal conditions, managed across primary, secondary, or community care settings. Studies evaluating the cost-effectiveness of AI as an intervention were included. For the review, AI refers to the science of creating intelligent machines that use algorithms to mimic human cognitive functions such as learning, reasoning, and problem-solving [19]. They are designed to analyse large, complex datasets, recognise patterns and relationships, and make predictions or decisions in an adaptive and often autonomous manner. Comparators include usual care or standard clinical management, non-AI digital interventions, alternative diagnostic or therapeutic strategies, or no intervention or no screening, depending on the study context. Outcomes of interest include economic outcomes such as total and incremental costs, cost-effectiveness or cost-utility ratios (e.g. cost per quality-adjusted life year), cost per clinical outcome achieved, net monetary benefit, or cost–benefit metrics, alongside relevant health outcomes including pain, disability, quality of life, healthcare utilisation, and productivity losses. Eligible study designs include full economic evaluations, such as cost-effectiveness, cost-utility, cost–benefit, and cost-consequences analyses, conducted using trial-based or model-based approaches with prospective, retrospective, or decision-analytic designs.

We excluded interventions without an AI component, studies not reporting economic outcomes, reviews, meta-analyses, protocols, letters, editorials, qualitative studies, conference abstracts, and articles not published in English.

### Data extraction and reporting transparency assessment

Data extraction was completed using a standardised form in Microsoft Excel. One reviewer (TG) extracted data, which were independently verified by two reviewers (FNO and CM). Extracted information included study characteristics (first author, year, country, sample size, study population, intervention, comparator, and time-horizon), type of economic evaluation, perspective, discount rate, cost data including direct and indirect costs (currency, and direct or indirect costs), health outcomes (QALYs, pain reduction, functional outcomes), and incremental cost-effectiveness ratios (ICERs).

The reporting transparency of the included studies was evaluated using the Consolidated Health Economic Evaluation Reporting Standards (CHEERS) checklist [20]. This checklist evaluates 28 key domains, including study design, analytic perspective, time horizon, outcome measurement, cost estimation, modelling assumptions, and uncertainty analyses. Each included study was independently appraised by two reviewers (TG and CM) to ensure consistency and accuracy. Discrepancies in scoring or interpretation were resolved through discussion with a third reviewer (FNO). The level of adherence to CHEERS criteria was summarised across studies as high, moderate, or low quality, reflecting the completeness and robustness of economic reporting. Findings were synthesised descriptively to identify common strengths, methodological limitations, and areas requiring improved standardisation in future cost-effectiveness studies of AI-based interventions for MSDs of the spine.

### Data analysis

Data analysis for this systematic review involved a structured synthesis of cost-effectiveness evidence across included studies. Extracted data were tabulated and compared in terms of study characteristics, intervention types, comparators, time horizons, perspectives (healthcare system or societal), and outcome measures such as ICERs. Due to the heterogeneity in methodologies, populations, and outcome reporting, a narrative synthesis has been conducted, supported by summary tables (Tables 1 and 2).

**Table 1:** Characteristics of the included studies

Reference, Country	Type of condition	Trial-based vs. model-based	Target population / sample Size	Mean age (years)	Time horizon	Type of economic evaluation	Intervention	Comparator
Park et al. [21], South Korea	LBP	RCT	100 participants (Int = 50; Cot = 50); Female/Male = 40/60	Int: 37.1 ± 8.0; Cot: 33.2 ± 6.1	4 weeks	CUA	Digital Application Physical Therapy (DPT), an AI-based smartphone-delivered rehabilitation program providing individualized exercise prescription with real-time audiovisual feedback,	Participants receiving conventional physical therapy (CPT), delivered as 30-minute in-person sessions three times per week for four weeks and including standard modalities such as heat, ultrasound, mobilization, manipulation, and therapeutic exercises
Reginster et al. [26], Germany	Osteoporosis / Osteoporotic fractures	Decision tree + microsimulation Markov model	German women aged 50+; 5,000,000 simulated individuals	Not explicitly reported; population includes women 50+ (subgroups 50–64 and 65+)	Lifetime horizon (up to age 100)	CUA	Opportunistic osteoporosis screening using AI-driven deep learning applied to routine chest radiographs, followed by risk-based clinical management. Women identified at elevated fracture risk received pharmacological treatment.	No screening and no treatment scenario, representing usual care in which women aged 50 years and older did not undergo AI-driven chest radiograph screening or subsequent osteoporosis treatment.
Kongstad et al. [16], Denmark	LBP	RCT	Int = 149; Cot = 148; Females: Int = 80 (53.7%), Cot = 84 (56.8%)	Int: 51.1 (15.2); Cot: 50.3 (13.8)	9 months	CUA/CEA	The intervention was an AI-based, app-delivered self-management programme (selfBACK) providing individually tailored advice on physical activity, exercises, and education as an add-on to usual care for people with low back pain.	Usual care alone, consisting of standard advice and treatment delivered by healthcare professionals in primary care or outpatient spine clinics.
Gorelik et al. [24], USA	Inflammatory back pain	Decision analytic model	N/A	N/A	3 years	CUA	Diagnostic imaging strategies for the initial detection of sacroiliitis in suspected axial spondyloarthritis, including radiography alone, MRI alone, and radiography followed by MRI if initial radiographs were negative.	Radiography alone of the sacroiliac joints.
Hornberger et al. [25], USA	Failed back surgery syndrome	Decision analytic model	N/A	N/A	Lifetime	CCA	Spinal cord stimulation (SCS) using a rechargeable implantable pulse generator for patients with failed back surgery syndrome	Spinal cord stimulation using a conventional non-rechargeable implantable pulse generator.
Morgan et al. [22], Australia	LBP	Observational study (retrospective analysis)	Population-level intervention; sample not specified	N/A	20 months	CEA/CBA	The AI element in the National Prescribing Service (NPS) MedicineWise LBP program is the Back Pain Choices online decision support tool, which uses a rule-based algorithm to guide GPs in diagnosing and managing acute LBP. A national educational and audit-and-feedback program	A no-intervention (usual practice) scenario.
Priebe et al. [23], Germany	LBP	RCT	Int = 930; Cot = 307	Int: 42.0 (12.4); Cot: 37.0 (12.6)	12 months	CEA	A digitally anchored, multimodal back pain management approach (Rise-uP) centred on a medical app (Kaia back pain app) delivering exercise therapy, mindfulness training, and education, supported by GP-led care and teleconsultations for high-risk patients.	Patients receiving standard of care for non-specific low back pain, delivered by general practitioners.

Int = Intervention, Cot = Control; LBP = Low back pain, RCT = Randomised controlled trial; CEA = Cost effectiveness analysis; CBA = Cost benefit analysis; CUA = Cost Utility Analysis; CCA=Cost-consequences analysis, N/A = Not available; GP = General Practitioners; NPS = National Prescribing Service, LBP = Low back pain

**Table 2:** Transparency scoring using the CHEERS criteria of the included studies

CHEERS Criterion	Summary of criterion	Studies meeting criterion *n* (%)	Studies not meeting criterion *n* (%)
1	Title	7 (100%)	0 (0%)
2	Abstract	7 (100%)	0 (0%)
3	Background and objectives	7 (100%)	0 (0%)
4	Target population and subgroups	7 (100%)	0 (0%)
5	Setting and location	6 (85.7%)	1 (14.3%)
6	Study perspective	6 (85.7%)	1 (14.3%)
7	Comparators	7 (100%)	0 (0%)
8	Time horizon	7 (100%)	0 (0%)
9	Discount rate	4 (57.1%)	3 (42.9%)
10	Choice of health outcomes	7 (100%)	0 (0%)
11a	Measurement of effectiveness – single study based	4 (100%)*	0 (0%)*
11b	Measurement of effectiveness – synthesis based	3 (100%)*	0 (0%)*
12	Measurement and valuation of preference-based outcomes	4 (57.1%)	3 (42.9%)
13a	Estimating resources and costs – single study based	4 (100%)*	0 (0%)*
13b	Estimating resources and costs – model based	3 (100%)*	0 (0%)*
14	Currency, price date and conversion	6 (85.7%)	1 (14.3%)
15	Choice of model	3 (100%)*	0 (0%)*
16	Assumptions	5 (71.4%)	2 (28.6%)
17	Analytic methods	7 (100%)	0 (0%)
18	Study parameters	7 (100%)	0 (0%)
19	Incremental costs and outcomes	7 (100%)	0 (0%)
20a	Characterising uncertainty – single study based	4 (100%)*	0 (0%)*
20b	Characterising uncertainty – model based	3 (100%)*	0 (0%)*
21	Characterising heterogeneity	2 (28.6%)	5 (71.4%)
22	Study findings, limitations, generalisability, current knowledge	7 (100%)	0 (0%)
23	Source of funding	6 (85.7%)	1 (14.3%)
24	Conflicts of interest	6 (85.7%)	1 (14.3%)

*Percentages for items 11, 13, 15 and 20 calculated using relevant denominators, consistent with CHEERS guidance

## Results

We identified 347 records in Web of Science (*n* = 95); MEDLINE & CINAHL (*n* = 17); Cochrane Central Register of Controlled Trials (*n* = 113), PubMed (*n* = 8), Scopus (*n* = 87) and DOAJ (*n* = 27) databases. Of these records, 59 were duplicates. Following screening by titles and abstracts, 251 studies were excluded, leaving 37 articles for full-text review. After reading the full text of these articles, only 7 met the inclusion criteria and were eligible for this systematic review. No additional article was identified from the searches of Scopus and the Directory of Open Access Journals (DOAJ) to be included in the review.

### Characteristics of the included studies

A total of seven studies published between 2008 and 2025 were included in this review, representing diverse geographical settings (South Korea, Denmark, United States, Australia, and Germany). The majority of studies focused on LBP, either chronic or non-specific [16, 21–23], while one evaluated inflammatory back pain [24], and another addressed failed back surgery syndrome and Osteoporosis [25, 26].

### Population characteristics

Mean ages of RCT participants ranged from the mid-30s to early 50s. Park et al. [21] included younger adults with chronic LBP (mean 35.5 years), whereas Kongstad et al. [16] and Priebe et al. [23] recruited broader adult populations (mean ages 42–51 years). Gender distribution was generally balanced, though some variation existed across trials.

### Type of economic evaluation

Across the seven included studies, multiple types of economic evaluation were identified. Three studies conducted cost-utility analyses (CUA) using quality-adjusted life years as the primary outcome [21, 24, 26]. One study employed a cost-consequences analysis (CCA), reporting costs and outcomes separately within a decision-analytic model [25]. One study applied a combined economic evaluation approach, undertaking a trial-based cost-utility analysis as the primary evaluation alongside additional cost-effectiveness analyses (CEA) using disease-specific clinical outcomes [16]. Another study used both cost-benefit analysis (CBA) and cost-effectiveness analysis, evaluating a national low back pain program by estimating monetary costs and savings and reporting the cost per CT scan averted [22].

### Perspective, time horizon, trial-based vs. model-based

The included studies varied in design, time horizon, and analytic perspective. Three studies reported trial-based economic evaluations conducted alongside randomised controlled trials, with follow-up periods ranging from four weeks [21] to nine months [16] and twelve months [23]. Four studies used non–trial-based approaches, including three decision-analytic or Markov models [24; 25; 26] and one observational post-implementation evaluation [22].

Time horizons varied substantially across the included studies. Short-term horizons were applied in trial-based evaluations, including four weeks [21], nine months [16], and twelve months [23]. Longer horizons were used in model-based studies, including three years [24] and lifetime horizons extending to death or age 100 [25, 26]. The observational study assessed costs over a 20-month post-implementation period [22].

Analytic perspectives differed across studies. Two studies [21, 24] adopted healthcare or health system perspective. A payer perspective was reported by Reginster et al. [26], while a government department perspective was used by [22]. Three studies reported a societal perspective [16, 23, 25].

### Comparator categories

Across the included studies, control categories varied substantially, reflecting differences in intervention type and evaluation design. Several trial-based studies used usual care or standard clinical management as the comparator, such as conventional physical therapy [21] and guideline-based primary care without digital support [16, 23]. Model-based evaluations commonly employed no screening or no intervention scenarios, representing routine practice without AI-enabled tools [24, 26]. Device-based comparisons contrasted AI-enabled or advanced technologies with conventional alternatives, such as rechargeable versus non-rechargeable systems in Hornberger et al. [25]. Program-level evaluations used observational or counterfactual controls, including non-exposed provider groups or modeled “no program” scenarios [22].

### Intervention modalities

Across the included studies, intervention modalities and types varied widely, reflecting the breadth of AI and digital health applications relevant to spine and LBP related conditions. Several studies evaluated digital, AI-enabled self-management or rehabilitation interventions, including app-based or software-driven programs designed to support exercise, education, and behaviour change alongside usual care [16, 21, 23]. Other studies focused on AI-supported diagnostic or decision-support modalities, such as imaging strategies or screening tools intended to improve diagnostic accuracy or optimize care pathways [24, 26]. At a system level, one study assessed a multifaceted implementation intervention, combining decision support, audit and feedback, and educational tools to influence clinician behaviour [22]. In contrast, one study compared technology-enabled devices with conventional alternatives within a decision-analytic framework [25].

### Reporting transparency of included studies

The CHEERS reporting transparency assessment shows that the overall reporting transparency of the seven included economic evaluations was high, with consistent compliance across key domains including background and objectives, comparators, time horizon, analytic methods, study parameters, and reporting of incremental costs and outcomes. Most included studies also adequately reported setting, perspective, costing methods, uncertainty analyses, funding sources, and conflicts of interest, particularly among model-based evaluations [22, 24, 26]. However, explicit reporting of discount rates was inconsistent especially in short-horizon trial-based studies where discounting was not required but should have been stated and analyses of heterogeneity were rarely undertaken.

Across the seven included studies, uncertainty was generally addressed in a systematic but variable manner, reflecting differences in study design and analytic approach. Trial-based economic evaluations primarily characterised uncertainty using non-parametric bootstrapping, confidence intervals, and cost-effectiveness planes, allowing assessment of joint uncertainty around costs and effects [16, 21, 23]. Model-based studies employed more extensive approaches, including deterministic one-way sensitivity analyses, probabilistic sensitivity analyses, and scenario analyses, to test the robustness of results to key assumptions such as time horizon, discount rates, adherence, device longevity, and costs [22, 24–26]. Despite these strengths, uncertainty related to structural assumptions, long-term extrapolation, and heterogeneity across patient subgroups was less consistently explored, and only a small number of studies conducted formal subgroup or scenario analyses to address these dimensions.

### Key findings of the included studies

Table 3 illustrates substantial variability in the cost-effectiveness of musculoskeletal AI interventions according to intervention type, analytical perspective, and outcome assessed. From a healthcare system perspective, several interventions demonstrate favourable economic performance. Digital physical therapy (DPT) was found to be cost saving and marginally more effective than conventional physical therapy, resulting in a dominant economic profile driven by reduced costs and small QALY gains [21]. In contrast, more intensive imaging strategies, including MRI alone or in combination with radiography, were associated with markedly higher costs and only modest improvements in QALYs, leading to very high ICERs and indicating a lack of cost-effectiveness [24]. Studies adopting both healthcare and societal perspectives reported modest increases in costs alongside measurable health benefits [16]. Specifically, the selfBACK app increased costs under both perspectives but achieved a statistically significant QALY gain, yielding ICERs within accepted willingness-to-pay thresholds and a high probability of being cost-effective [16].

**Table 3:** Summary of cost-effectiveness findings of the included studies

Reference	Perspective	Discount rate	Intervention / Comparator	Incremental Cost	Incremental Effect	ICER / Cost-Effectiveness	Conclusion
Park et al. [21]	Healthcare system	No discount rate	DPT vs. Conventional PT (CPT)	–US$113 (savings)	QALY gain (0.09 vs. 0.089)	Dominant (less costly, more effective)	DPT is cost-saving and more effective.
Kongstad et al. [16]	Healthcare & Societal	No discount rate	selfBACK app vs. usual care	€230 (healthcare); €589 (societal)	+ 0.03 QALY (95% CI 0.01–0.05)	€7,336 (healthcare); €18,821 (societal)	Cost-effective; 98% probability under €23,250 threshold.
Gorelik et al. [24]	Healthcare system	3% per annum	Radiography vs. MRI vs. combined	MRI: US$30,784 ↑; Combined: US$48,894 ↑	+ 0.05–0.08 QALY	MRI: US$569,792/QALY; Combined: US$569,742/QALY	Not cost-effective; radiography alone preferred.
Hornberger et al. [25]	Societal	3% per annum	Rechargeable SCS vs. non-rechargeable SCS	Lifetime savings: US$104,000–168,833	Fewer replacements (2.6–4.2 fewer)	Dominant (cost-saving)	Rechargeable SCS reduces long-term costs significantly.
Morgan et al. [22]	Government (health dept.)	5% per annum	NPS MedicineWise vs. no intervention	AUD$11.6 M savings to MBS	10.85% ↓ in CT scans	$2.82 per CT scan averted	Policy intervention reduces unnecessary imaging, major savings (saved $11.6 million in MBS costs)
Reginster et al. [26]	German Statutory Health Insurance (SHI)	3% per annum	AI-driven chest radiograph screening vs. no treatment	€39 per woman	+ 0.0029 QALYs per woman; 4.4 fractures prevented per 1,000 women	€13,340 per QALY gained; dominant in women 50–64 or with improved adherence	AI-driven opportunistic screening is cost-effective and can improve early osteoporosis detection.
Priebe et al. [23]	Societal	No discount rate	Rise-uP program vs. control	€372.45 ↓ (per patient)	Better satisfaction (25.38 vs. 21.56); Pain ↓	€416.21 saved per NRS pain point reduction	Rise-uP yields cost savings and improved outcomes.

MRI = Magnetic Resonance Imaging; N/A = Not available; NPS = National Prescribing Service; AUD = Australian Dollar; DPT = Digital Physical therapy; QALY = Quality-adjusted life year; MBS = Medicare Benefits Schedule; NRS = Numeric rating scale

From a societal perspective, several interventions were associated with both cost savings and improved outcomes. Rechargeable spinal cord stimulation systems generated substantial lifetime cost savings by reducing the need for device replacements, resulting in a dominant economic profile [25]. Similarly, the Rise-uP program was shown to reduce per-patient costs while improving patient satisfaction and pain outcomes, yielding clear savings per unit of pain reduction [23]. At the policy and system level, a government-led educational intervention significantly reduced unnecessary CT imaging, leading to substantial overall savings in health expenditure at a low cost per scan averted [24]. AI-driven opportunistic screening for osteoporosis incurred small additional per-person costs but produced QALY gains and fracture prevention benefits, resulting in a cost-effective ICER and dominance in specific subgroups [26].

## Discussion

To best of our knowledge this is the first systematic review to specifically examine the cost-effectiveness of AI-based interventions in rheumatology. While economic evaluations interventions for MSDs and digital health solutions have been explored previously, to date, no prior synthesis has focused on AI-driven modalities as a central intervention strategy [27]. By collating evidence from RCTs, observational research, and decision-analytic modelling, this review provides an original contribution to the literature by highlighting how AI-enhanced approaches are reshaping both the clinical and economic landscape of MSDs management.

Across the seven included studies, the findings indicated a substantial heterogeneity on patterns that mirror those commonly observed in evaluations of the cost-effectiveness of AI-based interventions for MSDs. Digital health solutions such as AI-supported exercise prescription platforms [21], AI-driven chest radiograph screening [26], AI-supported self-management applications [16], and algorithm-driven care pathways [23] consistently reported favourable cost-effectiveness when compared with usual care or standard physiotherapy. These results align with recent systematic reviews of digital self-management programmes for low back pain, which have demonstrated improvements in patient-reported outcomes alongside potential cost savings through reduced healthcare utilisation and fewer in-person consultations [28].

Increasingly, the integration of AI into digital self-management programmes (SMPs) is recognised as a key mechanism for reducing costs, enhancing treatment adherence, and supporting the long-term sustainability of care delivery [29]. AI-enabled tools not only show promise in lowering direct healthcare costs, but also in mitigating certain indirect costs through improved efficiency and reduced need for in-person care. However, a comprehensive economic perspective must also account for the often substantial upfront and ongoing indirect costs associated with implementation. These include initial development and deployment expenses, investments in digital infrastructure, data storage and interoperability systems, and stringent data security and governance requirements [30]. In addition, the training and upskilling of healthcare professionals to effectively use and interpret AI-driven tools represent a significant resource commitment [31].

Despite these challenges, AI-driven SMPs can enhance patient engagement by offering continuous feedback, personalised goal setting, and adaptive treatment pathways. By leveraging real-time data and predictive analytics, these platforms can anticipate patient needs, optimise resource allocation, and reinforce adherence to evidence-based interventions. Over time, such efficiencies may offset initial investments, highlighting AI’s emerging role in delivering personalised, scalable, and economically efficient models of care that align clinical effectiveness with sustainable resource use [32, 33].

The cost-effectiveness of AI interventions for MSDs across the three included studies [21, 24, 26] varied widely, largely reflecting differences in clinical use and implementation costs. Although all interventions produced positive incremental QALYs, their economic impacts ranged from clear savings to substantial additional costs. AI-supported digital physical therapy [21] was cost-saving, whereas AI-assisted osteoporosis screening [26] added only minimal cost for small health gains. In contrast, MRI-based diagnostic AI [24] yielded moderate benefits but at very high additional expense.

Across the seven included studies, follow-up duration varied widely, from short-term trial-based follow-up of 4 weeks to 12 months to multi-year or lifetime modelling horizons, as well as a 20-month post-implementation evaluation. This variation is likely to influence estimated costs and outcomes, as shorter follow-up periods primarily capture immediate benefits of digital and AI-supported interventions, whereas longer horizons incorporate downstream effects and sustainability [21, 24, 26]. Evidence from studies with longer follow-up suggests that initial benefits may diminish or plateau over time, consistent with broader concerns regarding long-term adherence and maintenance of effectiveness in digital health interventions [16, 23]. However, no study explicitly assessed temporal correlations between follow-up duration and economic outcomes, such as by varying follow-up length within analyses, and this was not feasible due to heterogeneity in study designs, intervention modalities, outcome measures, and reliance on extrapolated assumptions in model-based evaluations.

The findings from the two model-based studies [24, 25] are broadly consistent with prior economic analyses that have projected favourable long-term outcomes for device-based or imaging-guided strategies [34]. Yet, the reliance on assumptions and lack of real-world validation in these studies contrast with current calls for empirical grounding and pragmatic evaluation of AI-enabled healthcare technologies [33]. Similarly, while the observational evidence from Morgan et al. [22] offered valuable real-world insights into system-level impacts, its vulnerability to confounding highlights a persistent limitation in the broader evidence base: a heavy reliance on retrospective analyses and simulation models, rather than large-scale prospective trials [32, 35].

The results of the current review suggest that AI-enabled interventions hold considerable economic potential, particularly for chronic and non-specific LBP, consistent with wider trends in digital health research [32, 34]. Nonetheless, the evidence remains fragmented and methodologically diverse, contrasting with the more robust and standardised evaluation frameworks that are increasingly advocated for in health technology assessment [33]. This tension between early promise and evidentiary gaps reflects the current state of knowledge: AI interventions are progressing rapidly in practice, yet economic evaluation methods have not kept pace, leaving a critical need for more rigorous, comparative, and long-term studies [36, 37].

**Fig. 1:**
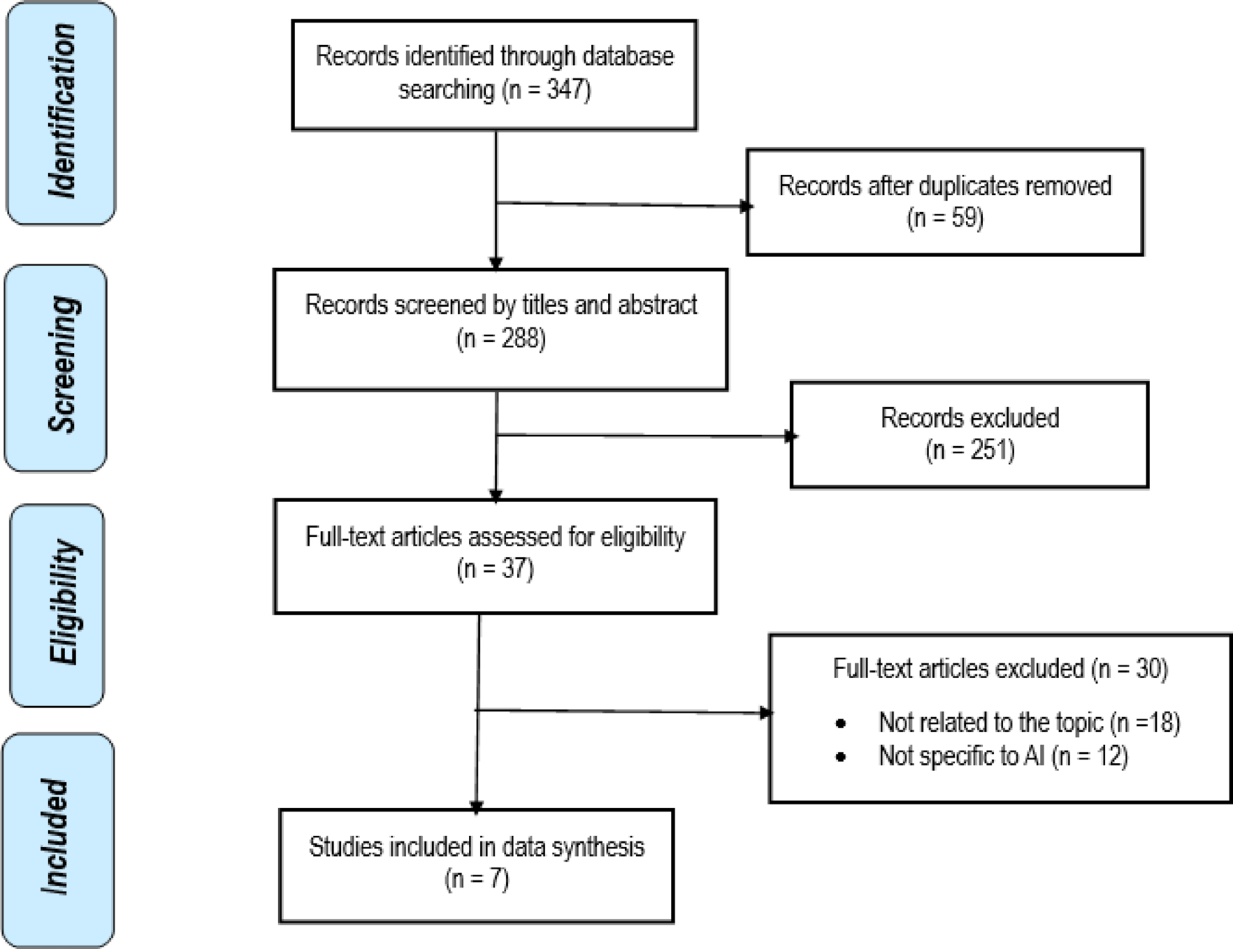
Flow diagram of publications included and excluded in the review

There are notable strengths of the present review. The inclusion of multiple study designs offered a broad perspective on AI applications, spanning both clinical trials and health system analyses. The geographical diversity of studies, conducted in Asia, Europe, North America, and Australia, adds to the international relevance of findings. The inclusion of follow-up periods ranging from short-term trial data to lifetime modelling horizon offers a comprehensive perspective on both immediate and long-term cost-effectiveness. Furthermore, methodological rigour of the review was assured by the use PRISMA which is standard guidelines for reporting systematic reviews, the CHEERS for assessing methodological quality of the included studies. This ensures a transparent, thorough, and unbiased synthesis of the available evidence (Fig. 1).

The clinical implication of the review is that integrating AI-based interventions into routine management for MSDs could support more efficient, value-based decision-making to improve patient outcomes while reducing healthcare costs. This suggests clinicians and healthcare systems are to consider adopting such technologies to optimize resource use without compromising and potentially enhancing quality of care [38].

Across the seven included studies, several factors should be considered when interpreting the findings, including publication bias, methodological quality, and heterogeneity of results. First, publication bias is likely, as all included studies reported cost-saving or cost-effective findings, with no clearly negative economic evaluations, suggesting that studies demonstrating favourable results may be more likely to be published. Second, while the overall methodological and reporting transparency of the studies was high according to the CHEERS assessment, recurrent limitations were identified, particularly inconsistent reporting of discounting decisions and limited exploration of heterogeneity, which may affect transparency and reproducibility. Third, the currently available evidence included in present review is predominantly focused on low back pain and spine-related conditions, which may limit the generalisability of findings across the broader spectrum of MSDs. Finally, there was substantial heterogeneity across studies in terms of intervention types (digital therapies, imaging strategies, implantable devices, policy interventions, and AI screening), analytic perspectives (healthcare, societal, and mixed perspectives), time horizons, outcome measures (QALYs, pain scores, imaging rates), and modelling approaches, which resulted in widely varying cost and effectiveness estimates. This heterogeneity precludes meaningful quantitative synthesis and limits cross-study comparability, reinforcing the need for cautious interpretation of results and for greater methodological standardisation in future economic evaluations.

## Conclusion

The findings of this review suggest that AI-based interventions have the potential to reduce the economic burden MSDs of the spine while improving health outcomes. However, given the heterogeneity of the available evidence, these results should be interpreted with caution. Although such interventions could contribute to more efficient allocation and use of healthcare resource, and inform policy or reimbursement decisions, further methodologically consistent economic evaluations are needed to strengthen the evidence base.

## Supplementary Information

Below is the link to the electronic supplementary material.

Supplementary Material 1

## Data Availability

All data analysed during this study are included in this published article and its supplementary information files. No new data were generated.

## Declarations

### AI use statement

The authors declare that no artificial intelligence (AI) tools were used in the design of the study, data collection, data analysis, or interpretation of the results. AI tools were used only for language editing and improvement of readability, and all content has been carefully reviewed and approved by the authors, who take full responsibility for the integrity and accuracy of the work.

## References

[1] Vos T, Lim SS, Abbafati C, Abbas KM, Abbasi M, Abbasifard M, Abbasi-Kangevari M, Abbastabar H, Abd-Allah F, Abdelalim A, Abdollahi M (2020) Global burden of 369 diseases and injuries in 204 countries and territories, 1990–2019: a systematic analysis for the Global Burden of Disease Study 2019. 10.1016/S0140-6736(20)30925-9. Lancet 10.1016/S0140-6736(20)30925-9 PMC7567026 33069326

[2] Wu A, March L, Zheng X, Huang J, Wang X, Zhao J, Blyth FM, Smith E, Buchbinder R, Hoy D (2020) Global low back pain prevalence and years lived with disability from 1990 to 2017: estimates from the Global Burden of Disease Study 2017. 10.21037/atm.2020.02.175. Ann Transl Med 10.21037/atm.2020.02.175 PMC7186678 32355743

[3] Oliveira CB, Maher CG, Pinto RZ, Traeger AC, Lin CW, Chenot JF, van Tulder M, Koes BW (2018) Clinical practice guidelines for the management of non-specific low back pain in primary care: an updated overview. Eur Spine J. 10.1007/s00586-018-5673-2 29971708 10.1007/s00586-018-5673-2

[4] Qaseem A, Wilt TJ, McLean RM, Forciea MA (2017) Noninvasive treatments for acute, subacute, and chronic low back pain: a clinical practice guideline from the American College of Physicians. 10.7326/M16-2367. Clinical Guidelines Committee of the American College of PhysiciansAnn Intern Med 10.7326/M16-2367 28192789

[5] Hayden JA, Ellis J, Ogilvie R, Malmivaara A, van Tulder MW (2021) Exercise therapy for chronic low back pain. Cochrane Database Syst Rev. 10.1002/14651858.CD009790.pub2 34580864 10.1002/14651858.CD009790.pub2 PMC8477273

[6] Sanabria-Mazo JP, Colomer-Carbonell A, Fernández-Vázquez Ó, Noboa-Rocamora G, Cardona-Ros G, McCracken LM, Montes-Pérez A, Castaño-Asins JR, Edo S, Borràs X, Sanz A (2023) A systematic review of cognitive behavioral therapy-based interventions for comorbid chronic pain and clinically relevant psychological distress. Front Psychol. 10.3389/fpsyg.2023.1200685 38187407 10.3389/fpsyg.2023.1200685 PMC10766814

[7] Chen BL, Guo JB, Zhang HW, Zhang YJ, Zhu Y, Zhang J, Hu HY, Zheng YL, Wang XQ (2018) Surgical versus non-operative treatment for lumbar disc herniation: a systematic review and meta-analysis. Clin Rehabil. 10.1177/0269215517709887 28715939 10.1177/0269215517719952

[8] Montemurro N, Zotti N, Guercini J, De Carolis G, Leoni C, Marotta R, Tomei R, Baggiani A, Paolicchi A, Lazzini S, Di Serafino F (2024) Value-based healthcare in management of chronic back pain: a multidisciplinary- and lean-based approach. Surg Neurol Int. 10.25259/SNI_348_2024 39373005 10.25259/SNI_468_2024 PMC11450888

[9] Jawed AM, Zhang L, Zhang Z, Liu Q, Ahmed W, Wang H (2025) Artificial intelligence and machine learning in spine care: advancing precision diagnosis, treatment, and rehabilitation. World J Orthop. 10.5312/wjo.v16.i8.107064 40838224 10.5312/wjo.v16.i8.107064 PMC12362650

[10] Esteva A, Robicquet A, Ramsundar B, Kuleshov V, DePristo M, Chou K (2019) A guide to deep learning in healthcare. Nat Med. 10.1038/s41591-018-0316-z 30617335 10.1038/s41591-018-0316-z

[11] Khan O, Badhiwala JH, Grasso G, Fehlings MG (2020) Use of machine learning and artificial intelligence to drive personalized medicine approaches for spine care. World Neurosurg. 10.1016/j.wneu.2020.06.039 32797983 10.1016/j.wneu.2020.04.022

[12] Marcuzzi A, Nordstoga AL, Bach K, Aasdahl L, Nilsen TIL, Bardal EM (2023) Effect of an artificial intelligence-based self-management app on musculoskeletal health in patients with neck and/or low back pain referred to specialist care: a randomized clinical trial. JAMA Netw Open. 10.1001/jamanetworkopen.2023.20400 37368401 10.1001/jamanetworkopen.2023.20400 PMC10300712

[13] Toelle TR, Utpadel-Fischler DA, Haas KK, Priebe JA (2019) App-based multidisciplinary back pain treatment versus usual care: a randomized controlled trial. NPJ Digit Med. 10.1038/s41746-019-0109-x 31304380 10.1038/s41746-019-0109-x PMC6550294

[14] Park J, Kim S, Lee S (2019) Development and validation of an artificial intelligence-based system for spinal movement analysis in patients with low back pain. J Orthop Res. 10.1002/jor.2435631709604

[15] Gupta L, Najm A, Kabir K, De Cock D (2023) Digital health in musculoskeletal care: where are we heading? 10.1186/s12891-023-06234-3. BMC Musculoskelet Disord 10.1186/s12891-023-06309-w PMC10012296 36918856

[16] Kongstad LP, Øverås CK, Sandal LF (2024) Cost-effectiveness analysis of app-delivered self-management support for low back pain (selfBACK). BMJ Open. 10.1136/bmjopen-2023-086800 39242164 10.1136/bmjopen-2024-086800 PMC11381704

[17] Sandal LF, Bach K, Øverås CK (2021) Effectiveness of app-delivered, tailored self-management support for adults with low back pain–related disability: a selfBACK randomized clinical trial. JAMA Intern Med. 10.1001/jamainternmed.2021.5080 34338710 10.1001/jamainternmed.2021.4097 PMC8329791

[18] Priebe JA, Haas KK, Moreno Sánchez LF (2020) Real-world implementation aspects from the Rise-uP trial of app-supported back pain care: insights into adherence, patient pathways, and scalability. J Pain Res. 10.2147/JPR.S24744732765057

[19] Klingelhöfer D, Braun M, Dröge J, Groneberg DA, Brüggmann D (2025) Research on artificial intelligence, machine and deep learning in medicine: global characteristics, readiness, and equity. Glob Health. 10.1186/s12992-025-01128-1 10.1186/s12992-025-01128-1 PMC12147299 40484942

[20] Husereau D, Drummond M, Augustovski F, de Bekker-Grob E, Briggs AH, Carswell C, Caulley L, Chaiyakunapruk N, Greenberg D, Loder E, Mauskopf J, Mullins CD, Petrou S, Pwu RF, Staniszewska S (2022) Consolidated Health Economic Evaluation Reporting Standards 2022 (CHEERS 2022) statement. BMC Med. 10.1186/s12916-021-02211-4 35014160 10.1111/1471-0528.17012

[21] Park S, Lee J, Kim H (2023) Cost-effectiveness of an AI-driven exercise prescription platform for chronic low back pain: randomized controlled trial. J Med Internet Res. 10.2196/4512338032730

[22] Morgan T, Griffiths C, Turner J (2019) Economic evaluation of a feedback intervention to reduce imaging for low back pain in general practice. BMC Health Serv Res. 10.1186/s12913-019-4767-131864352

[23] Priebe J, Hauser W (2024) Cost-effectiveness of algorithm-based risk stratification and teleconsultation for low back pain in primary care: results from the Rise-uP pragmatic trial. Pain. 10.1097/j.pain.0000000000003058

[24] Gorelik A, Lee YC, Briggs AM (2020) Cost-effectiveness of imaging strategies for the assessment of inflammatory back pain. Arthritis Care Res (Hoboken). 10.1002/acr.23889

[25] Hornberger J, Kumar K, Verhulst E (2008) Rechargeable spinal cord stimulation versus non-rechargeable systems for patients with failed back surgery syndrome: cost–consequence analysis. Clin J Pain. 10.1097/AJP.0b013e31815ca2f2 18287831 10.1097/AJP.0b013e318160216a

[26] Reginster JY, Schmidmaier R, Alokail M, Hiligsmann M (2025) Cost-effectiveness of opportunistic osteoporosis screening using chest radiographs with deep learning in Germany. Aging Clin Exp Res. 10.1007/s40520-024-02786-4 40355760 10.1007/s40520-025-03048-x PMC12069426

[27] Fatoye F, Gebrye T, Mbada C, Useh U (2023) Economic evaluations of digital health interventions for the management of musculoskeletal disorders: systematic review and meta-analysis. J Med Internet Res 25:e41113 37410542 10.2196/41113 PMC10359913

[28] Fatoye F, Gebrye T, Odeyemi I (2023) Economic evaluations of digital health interventions for the management of musculoskeletal disorders: systematic review and meta-analysis. J Med Internet Res. 10.2196/47264 37410542 10.2196/41113 PMC10359913

[29] World Health Organization (2021) Global strategy on digital health 2020–2025. WHO

[30] Organisation for Economic Co-operation and Development (2019) Health in the 21st century: putting data to work for stronger health systems. OECD Publishing

[31] National Health Service (2019) The Topol Review: preparing the healthcare workforce to deliver the digital future. NHS

[32] Pawelczyk M, Muller S, Falla D (2025) Advancing musculoskeletal care using AI and digital health applications: a systematic review. J Clin Med. 10.3390/jcm1402051240565946

[33] Scala S (2024) App-based self-management strategies for low back pain: a systematic review and quality assessment. Life. 10.3390/life14060760 10.3390/life14060760 PMC11204566 38929744

[34] Klingenberg L, Kjaer P, Korsholm L (2023) The effect of therapeutic adherence on the effectiveness of a digital therapeutic exercise program for low back pain: cohort study. 10.3390/healthcare11192614. Healthcare

[35] Fatoye F, Gebrye T, Ryan CG, Useh U, Mbada C (2023) Global and regional estimates of clinical and economic burden of low back pain in high-income countries: systematic review and meta-analysis. Front Public Health. 10.3389/fpubh.2023.1098100 37383269 10.3389/fpubh.2023.1098100 PMC10298167

[36] Geraghty AWA, Little P, Foster NE (2024) Supporting self-management of low back pain with an internet intervention: 12-month outcomes of a randomised trial. Lancet Rheumatol. 10.1016/S2665-9913(24)00123-438824934

[37] Bartels A, Hildebrandt J, Lange J (2024) Digital approaches to chronic pain: a brief meta-review. Pain Manag. 10.2217/pmt-2023-0098

[38] Kongstad LP, Øverås CK, Skovsgaard CV, Sandal LF, Hartvigsen J, Søgaard K, Mork PJ, Stochkendahl MJ (2024) Cost-effectiveness analysis of app-delivered self-management support (selfBACK) in addition to usual care for people with low back pain in Denmark. BMJ Open. 10.1136/bmjopen-2024-086800 39242164 10.1136/bmjopen-2024-086800 PMC11381704

