# Supplementary material for: Cost-effectiveness of artificial intelligence interventions for musculoskeletal disorders of the spine: a systematic review
Source: Rheumatol Int. 2026 May 23;46(6):107. doi: 10.1007/s00296-026-06122-3 (PMC13198498; doi:10.1007/s00296-026-06122-3)
Supplement: Supplementary file 1 — Supplementary Material 1 [file 296_2026_6122_MOESM1_ESM.docx]

**Appendix 1: Search strategy**

S4 S1 AND S2 AND S3

S3 AB ("back pain" Or "neck pain" Or "spinal pain" Or "spin* pain" Or "thoracic pain" Or "cervical pain" Or "lumbar pain" Or "BACK PAIN" OR "LOW BACK PAIN" Or "NECK PAIN" Or "knee pain" Or "shoulder pain" Or "elbow pain" Or ''HAND pain'' Or ''WRIST pain'' OR "ankle pain" Or "foot pain" Or "hip pain" Or "musculoskeletal pain")

S2 AB (Cost-utility or cost utility or cost-benefit or cost benefit or cost-effectiveness or cost effectiveness or cost consequence or cost-consequence or economic outcome or economic evaluation or economic impact or health economic* or economic modelling or economic assessment)

S1 AB (''Machine Learning '' OR ''Deep Learning'' OR ''Clinical Decision Support System'' OR ''Natural Language Processing'' OR ''Computer-Aided Diagnosis'' OR ''Predictive Analytics'' OR ''Robotic Process Automation'' OR '' Digital Therapeutics'' OR ''Case-Based Reasoning'' OR ''Expert System'' OR ''Algorithmic Diagnosis'' OR '' Virtual Health Assistant Chatbot'' OR ''Artificial Intelligence'' OR ''AI'')
